# Ten-Year Trend of Retinopathy of Prematurity Among Extremely Preterm Infants in One Neonatal Intensive Care Unit in China

**DOI:** 10.3389/fped.2021.717090

**Published:** 2021-09-03

**Authors:** Yi Dai, Li Zhu, Yequn Zhou, Chao Chen, Shulian Zhang

**Affiliations:** Department of Neonatology, Children's Hospital of Fudan University, National Children's Medical Center, Shanghai, China

**Keywords:** extremely premature, trend, preterm infant, retinopathy of prematurity, incidence

## Abstract

**Background:** Extremely preterm (EP) infants are at the highest risk of retinopathy of prematurity (ROP). With more EP infants survived in China, recent data of ROP is lacking. The aim of the study is to report the trend of incidence of ROP among EP infants in a large neonatal intensive care unit in China over the past 10-year period, in relation with the overall survival rate and the change of oxygen saturation targets.

**Methods:** This retrospective cohort study enrolled all EP infants born before 28 weeks' gestation and admitted to one of the largest tertiary neonatal intensive care units in China from 2010 to 2019. Data were compared between two time periods according to different oxygen saturation targets: 2010–2014 (P1) with low saturation target and 2015–2019 (P2) with higher target.

**Results:** Of 630 EP infants admitted during the 10 years, 447 (71.0%) infants survived to discharge. The survival rate increased significantly from 61.6% in P1 to 75.8% in P2 (*P* < 0.05). Of the 472 infants who had ROP data, 318 (67.4%) developed ROP of any stage, 67 (14.2%) developed severe ROP, and 44 (9.3%) received treatment. The incidence of any ROP increased significantly from 51.7% in P1 to 74.3% in P2 (*P* < 0.05). The incidence of severe ROP increased from 11.0% in P1 to 15.6% in P2, and ROP treatment increased from 6.9% in P1 to 10.4% in P2, but neither reached statistical significance (both *P* > 0.05).

**Conclusions:** We observed an increasing trend in the incidence of ROP across the 10-year period in one of the largest neonatal care units in China. The increased survival rate and the use of high-target oxygen saturation in the later period may partly explain this trend. Further investigations are needed to improve the care practices and to reduce the incidence of severe ROP.

## Introduction

The survival rate of extremely preterm (EP, <28 weeks' gestation) infants in China has improved significantly during the last two decades. While with more EP infants survive, the incidences of major morbidities including severe retinopathy of prematurity (ROP) have been increasing (1). Severe ROP is the leading cause of childhood blindness worldwide (2). EP infants are at the highest risk of severe ROP and ROP requiring treatment. In a Swedish national cohort of EP infants, 9 of 434 (2.1%) were blind and 38 of 434 (8.8%) were visually impaired at 6.5 years, and visual problems were strongly associated with treated ROP (3). EP infants with severe ROP (stage ≥3 or requiring treatment) also have higher risks of cognitive and motor developmental delay, compared with those without severe ROP (4, 5).

ROP is a multifactorial disease, with prematurity and excessive oxygen exposure as the most important risk factors (6). Developed countries have experienced the “first epidemic (in the 1940s and 1950s)” and the “second epidemic (in the 1970s)” of ROP (7), and the incidence of severe ROP in EP infants began to decline or stabilize at a relatively low level (8). However, China, like other developing countries, is in the midst of “the third epidemic” of ROP (since 2000), with increased survival of EP infants and inadequate quality of neonatal care (9–11). Gilbert and colleagues called this “third epidemic” of severe ROP a mixture of first epidemic risk factors (inadequately monitored oxygen) and second epidemic risk factors (extreme prematurity) (7).Since the first guidance on oxygen therapy and the prevention and treatment of ROP issued by the Ministry of Health in 2004 (12), the prevention and management of ROP has improved considerably in China. A national multicenter study from 2010 to 2012 showed that the incidences of ROP and severe ROP in premature infants <34 weeks in China were 15.2 and 1.2%, respectively; and among infants with GA <28 weeks, the incidences of ROP and severe ROP were much higher, at 67.1 and 13.8%, respectively (13). However, during the past decade, there has been evolving targets of oxygen saturations for EP infants, which might influence incidence of ROP. In 2014, a meta-analysis and systematic review of several large trials assessing target oxygen saturation in EP infants showed that higher oxygen saturation targets (91–95% compared with 85–89%) were associated with decreased mortality (14). Based on this, many neonatal intensive care units (NICUs) in China adopted higher oxygen saturation target, though the same meta-analysis also showed increased risk of ROP associated with the higher saturation. Currently, limited data on the temporal trends of ROP incidence among EP infants are available in recent years from China.

The aim of the present study was to determine the trend of incidence and severity of ROP among EP infants in one of the largest NICUs in China during a 10-year period, in relation with the overall survival rate and the change of oxygen saturation targets.

## Materials and Methods

### Study Design, Setting, and Patients

This retrospective cohort study included all infants with gestational age <28 weeks and discharged between January 1, 2010 and December 31, 2019 from a tertiary NICU in Shanghai, China. Our NICU is one of the largest referral center for critical neonates in Shanghai and China with around 1,500 admissions annually. Our hospital is a free-standing children's hospital and all infants admitted to our unit are outborns. The study was approved by the Ethics Committee of the Children's Hospital of Fudan University and performed in agreement with the ethical principles in the Declaration of Helsinki.

From 2010 to 2014, our unit used the saturation target of 85%-89% for EP infants, and the target was changed to 91–95% from 2015. Therefore, the 10-year study period were divided into two phases: 2010–2014 (P1) and 2015–2019 (P2). The incidences and severities of ROP were compared between P1 and P2. Data on maternal and infant characteristics, and NICU treatments related with ROP were collected based on previous report of the study group (2).

### Diagnosis of ROP

ROP was assessed by qualified ophthalmologists with RetCam fundus camera. The diagnosis and categorization of ROP was made according to the revised International Classification of Retinopathy of Prematurity (IC-ROP) (15). The first fundus examination was performed at the 4 to 6 weeks after birth according to the national guideline issued in 2004 (12) and updated in 2014 (16). The indication for treatment was Type 1 pre-threshold ROP based on the Early Treatment of Retinopathy of Prematurity Study (ETROP) (17). The features of Type 1 pre-threshold ROP included any stage of ROP in zone 1 with plus, zone 1 stage 3 with or without plus, and zone 2 stage 2 or 3 with plus.

Severe ROP was defined as stage 3 or above or the need for treatment with laser or intravitreal anti-vascular endothelial growth factor (VEGF) therapy.

### Definitions

Gestational age was defined, in descending order of preference, from the early prenatal ultrasound result, last menstrual period, or New Ballard Score (18). Small for gestational age (SGA) was defined as birth weight (BW) less than the 10th percentile according to Zhu et al. (19). Antenatal steroid use was defined as any administration prior to birth, regardless of the time interval. Prolonged mechanical ventilation (MV) was defined as >7 days of invasive ventilation.

### Statistical Analysis

Results were presented as mean with standard deviation (SD), median with interquartile range (IQR), or numbers with percentage, as appropriate. Infants' characteristics were compared between P1 and P2 using the chi-square test for categorical variables and Student's *t*-test or the Mann-Whitney *U*-test for continuous variables. The trend analysis was performed with a modified Poisson regression model. SPSS statistical software (SPSS 20.0, SPSS Inc., Chicago, IL, USA) was used for analysis of the data. *P-*values were 2-tailed, and *P* < 0.05 was considered statistically significant.

## Results

**Figure 1:**
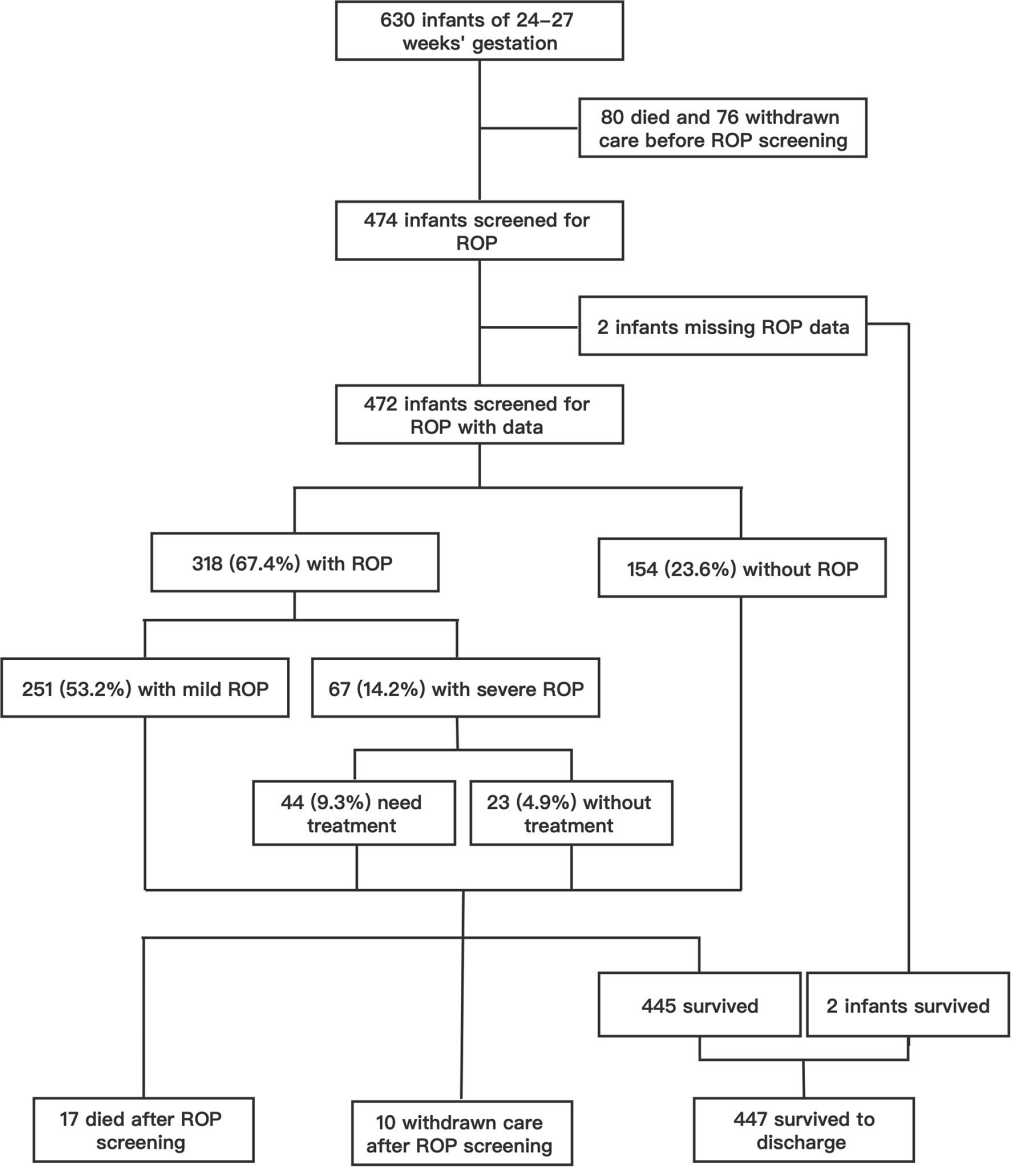
The flow chart of study infants and the incidence of ROP in patients who screened for ROP.

Over the 10 years from 2010 to 2019, a total of 630 infants born before 28 weeks' gestation were admitted to our NICU, with 216 admitted during P1 (2010–2014) and 414 during P2 (2015–2019). Of these, 97 (15.4%) infants died with active treatment, and 86 (13.7%) were taken home by their parents against medical advice. A total of 447 infants survived to discharge, with an overall survival rate of 71.0%. A total of 474 (75.2%) survived to the 5th week after birth and met ROP screening criteria and two infants had incomplete ROP data (Figure 1). Of the 472 infants with ROP screening and complete ROP data, the mean gestational age was 26.9 (SD 0.8) weeks, and mean birth weight was 1,017 (SD 156) g. ROP was found in 67.4% (318/472) of the infants, and severe ROP in 14.2% (67/472) (Figure 1). Overall, 44 (9.3%) received ROP treatment, including 28 infants who underwent laser therapy and 16 infants who were given an anti-VEGF drug (Ranibizumab) via intraocular injections (Figure 1).

**Figure 2:**
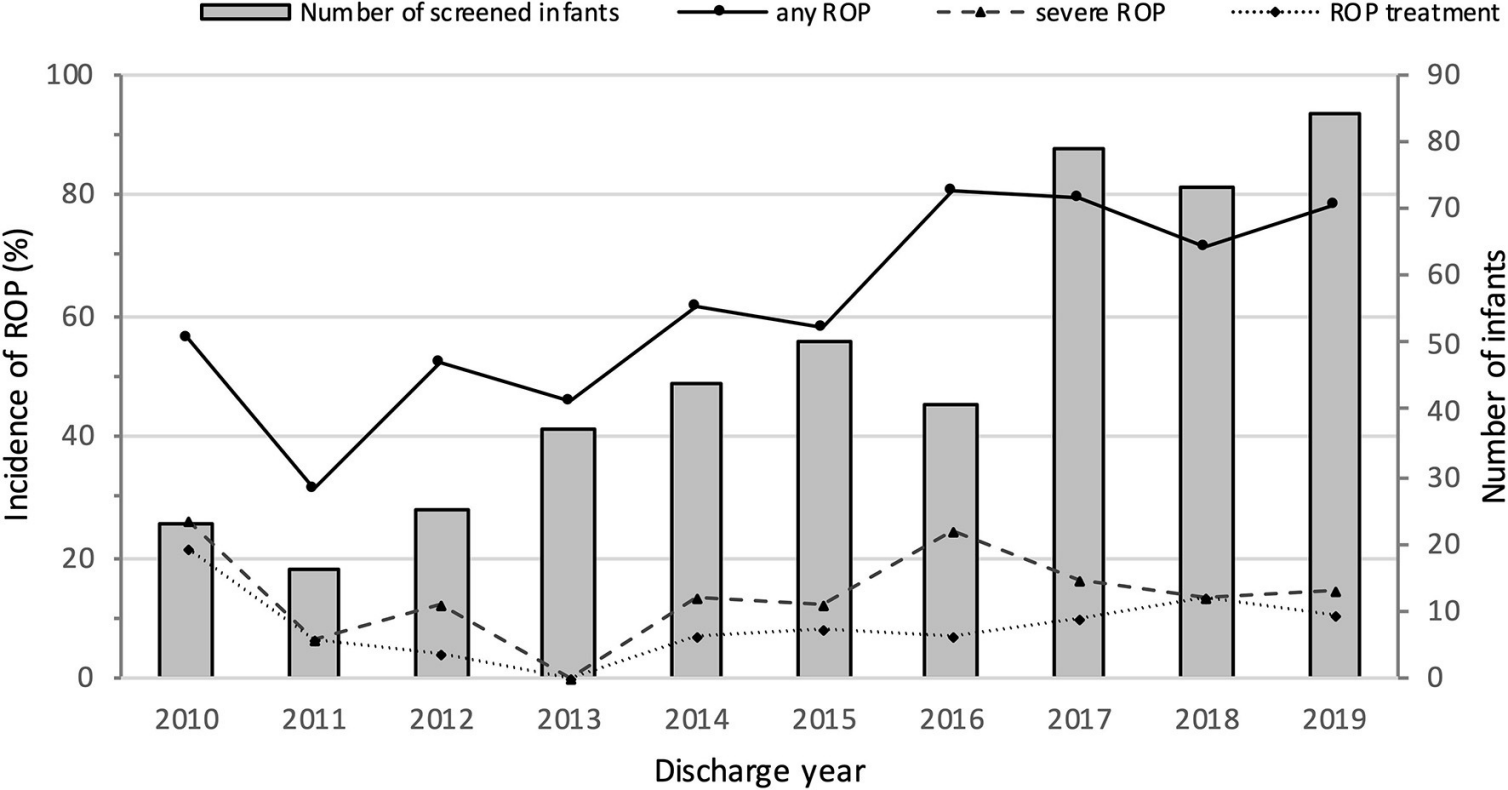
Numbers of screened infants and incidence of ROP in extremely premature infants from 2010 to 2019.

Mortality, survival, rates of ROP screening and incidences of ROP from 2010 to 2019 are shown in Table 1 and Figure 2. The number of EP infants increased from 34 in 2010 to 100 in 2019. The overall survival rate was 71.0%, and increased from 61.8% in 2010 to 78.0% in 2019 (*P* = 0.000). The number of infants who were screened for ROP increased from 23 in 2010 to 83 in 2019, with an increasing trend of ROP screening rate (*P* = 0.000). The incidence of any ROP ranged from 56.5% in 2010 to 78.6% in 2019, with a significantly increasing trend over the 10 years (*P* = 0.000). There were no significant trend observed in the incidence of severe ROP or ROP treatment (*P* = 0.603 and *P* = 0.448, respectively).

**Table 1:** Rates of mortality, withdrawn care, survival, and ROP in extremely preterm infants from 2010 to 2019^a^.

**Variable**	**2010 (*n* = 34)**	**2011 (*n* = 34)**	**2012 (*n* = 40)**	**2013 (*n* = 53)**	**2014 (*n* = 55)**	**2015 (*n* = 65)**	**2016 (*n* = 61)**	**2017 (*n* = 94)**	**2018 (*n* = 94)**	**2019 (*n* = 100)**	**Total (*n* = 630)**	***P*-value^c^**
Mortality	10 (29.4)	10 (29.4)	8 (20.0)	13 (24.5)	13 (23.6)	8 (12.3)	7 (11.5)	7 (7.4)	12 (12.8)	9 (9.0)	97 (15.4)	0.000
Withdrawn care	3 (8.8)	8 (23.5)	8 (20.0)	4 (7.5)	6 (10.9)	8 (12.3)	15 (24.6)	9 (9.6)	12 (12.8)	13 (13.0)	86 (13.7)	0.640
Survival	21 (61.8)	16 (47.1)	24 (60.0)	36 (67.9)	36 (65.5)	49 (75.4)	39 (63.9)	78 (83.0)	70 (74.5)	78 (78.0)	447 (71.0)	0.000
Screened for ROP	23 (67.6)	17 (50.0)	25 (62.5)	37 (69.8)	44 (80.0)	50 (76.9)	41 (67.2)	80 (85.1)	73 (77.7)	84 (84.0)	474 (75.2)	0.000
Screened and with data	23 (67.6)	16 (47.1)	25 (62.5)	37 (69.8)	44 (80.0)	50 (76.9)	41 (67.2)	79 (84.0)	73 (77.7)	84 (84.0)	472 (74.9)	0.000
Any ROP^b^	13 (56.5)	5 (31.3)	13 (52.0)	17 (45.9)	27 (61.4)	29 (58.0)	33 (80.5)	63 (79.7)	52 (71.2)	66 (78.6)	318 (67.4)	0.000
Severe ROP^b^	6 (26.1)	1 (6.2)	3 (12.0)	0 (0.0)	6 (13.6)	6 (12.0)	10 (24.4)	13 (16.5)	10 (13.7)	12 (14.3)	67 (14.2)	0.603
ROP treatment^b^	5 (21.7)	1 (6.2)	1 (4.0)	0 (0.0)	3 (6.8)	4 (8.0)	3 (7.3)	8 (10.1)	10 (13.7)	9 (10.7)	44 (9.3)	0.448

*ROP, retinopathy of prematurity*.

a *Data were shown as n (%)*.

b *Among infants who screened and with ROP data*.

c *P-values were determined for trend over the decade using modified Poisson regression models*.

Table 2 shows the rates of survival, mortality and withdrawal care in two periods by GA. As GA increased, the overall survival rates increased from 45.8% among infants born at 24 weeks to 76.9% among infants born at 27 weeks, and the overall mortality decreased from 50.0 to 11.3% among infants born at 24–27 weeks. In the comparisons of the two periods, the survival rate increased from 61.6% in P1 to 75.8% in P2 (*P* < 0.05). However, when stratified by GA, significant differences were seen only between the 26- and 27-week GA groups.

**Table 2:** Rates of survival, mortality, and withdrawal care in P1 (2010–2014) and P2 (2015–2019) by gestational weeks, n (%).

	**Survival**			**Mortality**			**Withdrawal care**		
	**Total**	**P1**	**P2**	**Total**	**P1**	**P2**	**Total**	**P1**	**P2**
24 w	11 (45.8)	4 (44.4)	7 (46.7)	12 (50.0)	5 (55.6)	7 (46.7)	1 (4.2)	0 (0.0)	1 (6.7)
25 w	41 (54.7)	13 (56.5)	28 (53.8)	19 (25.3)	6 (26.1)	13 (25.0)	15 (20.0)	4 (17.4)	11 (21.2)
26 w	115 (68.9)	26 (50.0)	89 (77.4)^*^	25 (15.0)	15 (28.8)	10 (8.7)^*^	27 (16.2)	11 (21.2)	16 (13.9)
27 w	280 (76.9)	90 (68.2)	190 (81.9)^*^	41 (11.3)	28 (21.2)	13 (5.6)^*^	43 (11.8)	14 (10.6)	29 (12.5)
24–27 w	447 (71.0)	133 (61.6)	314 (75.8)^*^	97 (15.4)	54 (25.0)	36 (10.4)^*^	86 (13.7)	29 (13.4)	57 (13.8)

* *Comparisons between P1 and P2, P < 0.05*.

**Figure 3:**
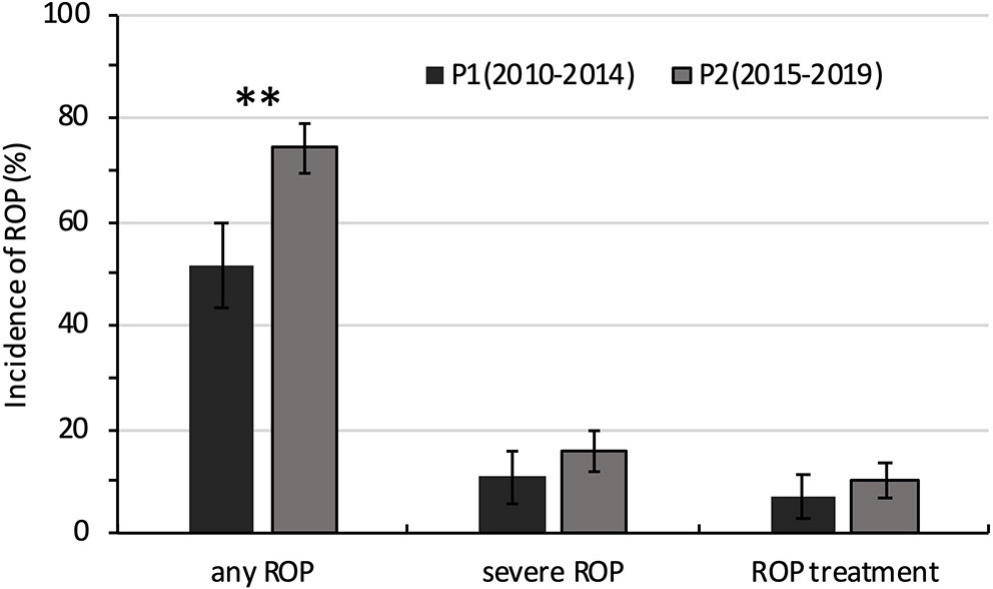
Comparison of the incidence of ROP in extremely premature infants between 2010–2014 (P1) and 2015−2019 (P2). **P < 0.05.

The incidence of any ROP and severe ROP increased with decreasing GA. The incidence of any ROP was significantly higher in P2 than in P1 (74.3 vs. 51.7%; *P* = 0.000) (Table 3, Figure 3). However, after GA stratification, differences were seen only between the 26- and 27-week GA groups. The incidence of severe ROP increased from 11.0% in P1 to 15.6% in P2, and ROP treatment increased from 6.9% in P1 to 10.4% in P2, but both increases were not statistically significant (*P* > 0.05) (Table 3, Figure 3).

**Table 3:** Rates of any ROP, severe ROP, and ROP treatment in 472 extremely preterm infants who completed ROP screening in P1 (2010–2014) and P2 (2015–2019) by gestational weeks, n (%).

	**Any ROP**			**Severe ROP**			**ROP treatment**		
	**Total**	**P1**	**P2**	**Total**	**P1**	**P2**	**Total**	**P1**	**P2**
24 w	11 (91.7)	5 (100.0)	6 (85.7)	5 (41.7)	0 (0.0)	5 (71.4)	3 (25.0)	0 (0.0)	3 (42.9)
25 w	43 (93.5)	12 (85.7)	31 (96.9)	14 (30.4)	4 (28.6)	10 (31.3)	11 (23.9)	3 (21.4)	8 (25.0)
26 w	90 (74.4)	13 (43.3)	77 (84.6)^*^	23 (19.0)	6 (20.0)	17 (18.7)	16 (13.2)	5 (16.7)	11 (12.1)
27 w	174 (59.4)	45 (46.9)	129 (65.5)^*^	25 (8.5)	6 (6.3)	19 (9.6)	14 (4.8)	2 (2.1)	12 (6.1)
24–27 w	318 (67.4)	75 (51.7)	243 (74.3)^*^	67 (14.2)	16 (11.0)	51 (15.6)	44 (9.3)	10 (6.9)	34 (10.4)

* *Comparisons between P1 and P2, P < 0.05*.

Table 4 presents the comparison of the basic characteristics and therapies among the 472 EP infants who completed ROP screening during the two periods. Mean GA and birth weight, proportions of male sex, SGA, and multiple birth remained similar in the two periods. There was no difference in the rates of maternal complications, including gestational diabetes mellitus, gestational hypertensive disease and premature rupture of membranes, between P1 and P2. The use of antenatal steroids increased from P1 to P2, as did the percentage of *in vitro* fertilization (IVF) and cesarean deliveries. Additionally, the use of caffeine and exclusive breastmilk significantly increased in the later period. Although there was no difference in the oxygen supplementation days between the two periods, the proportion of infants who required prolonged MV decreased significantly from P1 to P2. There was no significant difference in the proportions of infants who received blood transfusions three times or more in the two periods.

**Table 4:** Comparing basic characteristics and therapies in 472 extremely preterm infants who completed ROP screening in P1 (2010–2014) and P2 (2015–2019).

**Variable^a^**	**Total, *n* = 472**	**P1, *n* = 145**	**P2, *n* = 327**	***P-*value**
GDM	81 (17.2%)	18 (12.4%)	63 (19.3%)	0.069
Maternal hypertension	28 (5.9%)	6 (4.15)	22 (6.7%)	0.272
Antenatal steroid use	252 (53.4%)	53 (36.6%)	199 (60.9%)	0.000
IVF	177 (37.5%)	43 (29.7%)	134 (41.0%)	0.019
PROM	180 (38.1%)	54 (37.2%)	126 (38.5%)	0.79
Birth weight, g	1,017 (156)	1,022 (165)	1,015 (152)	0.659
GA, weeks	26.9 (0.8)	26.9 (0.9)	26.9 (0.8)	0.806
24 weeks GA	12 (2.5%)	5 (3.4%)	7 (2.1%)	0.405
25 weeks GA	46 (9.7%)	14 (9.7%)	32 (9.8%)	0.965
26 weeks GA	121 (25.6%)	30 (20.7%)	91 (27.8%)	0.101
27 weeks GA	293 (62.1%)	96 (66.2%)	197 (60.2%)	0.218
ELBW	200 (42.4%)	63 (43.4%)	137 (41.9%)	0.753
Male sex	288 (61.0%)	87 (60.0%)	201 (61.5%)	0.763
Cesarean delivery	109 (23.1%)	20 (13.8%)	89 (27.2%)	0.001
SGA	13 (2.8%)	6 (4.1%)	7 (2.1%)	0.358
Multiple births	193 (40.9%)	56 (38.6%)	137 (41.9%)	0.504
Blood transfusions ≥3	181 (38.3%)	49 (33.8%)	132 (40.4%)	0.175
Prolonged MV	211 (44.1%)	77 (53.1%)	134 (41.0%)	0.015
Oxygen days^b^	53 (40,72)	52 (41,73)	53 (40,70)	0.843
Exclusive EBM feeding	162 (34.3%)	1 (0.7%)	161 (49.2%)	0.000
Caffeine treatment	315 (66.7%)	13 (9.0%)	302 (92.4%)	0.000

*GDM, gestational diabetes mellitus; IVF, in vitro fertilization; PROM, premature rupture of membranes; GA, gestational age; ELBW, extremely low birth weight; SGA, small for gestational age; MV, mechanical ventilation; EBM, expressed breast milk*.

a *Categorical variables are expressed as n (%) and continuous variables as mean (SD) for normally distributed variables and median (IQR) for non-normally distributed variables*.

b *Oxygen days are summed up days from admissions*.

## Discussion

Our study analyzed the trend of ROP among EP infants in a tertiary NICU over a 10-year period. During this period, the distribution of gestational age and birth weight remained unchanged. ROP screenings were performed by experienced ophthalmologists using a RetCam fundus camera, and the indication for treatment was consistently based on the ETROP treatment threshold during the whole study period.

The overall incidences of any ROP and severe ROP in EP infants during 2010–2019 in our institute were 67.4 and 14.2%, which were similar to a multicenter study in China in 2010–2012. Compared with recently reported data on short-term outcome of EP infants in Guangdong Province during 2008–2017, the incidence was higher in our NICU for any ROP (67.2 vs. 45.1%), with similar incidences of severe ROP (14.2 vs. 14.6%); however, the survival rate was much higher in our NICU (74.9 vs. 52.5%).

In this 10-year period, we found an increasing trend of any ROP, and the incidence of ROP significantly increased from 51.7% in P1 to 71.3% in P2. The incidences for both severe ROP and treated ROP did not change significantly. However, though statistically unsignificant, the incidence of severe ROP increased by 41.8% from 11.0% in P1 to 15.6% in P2, and the incidence of ROP treatment increased by 50.7% from 6.9% in P1 to 10.4% in P2. Further longitudinal monitoring is required.

The incidence of severe ROP among EP infants in developed countries have been stabilized at a low level or showed decreasing trends. From 2007 to 2013, the overall incidence of ROP treatment in extremely premature infants in 11 International Network for Evaluating Outcomes (iNEO) member countries was 19.4% in 2007 and 13.7% in 2013, showing a general decreasing trend (8). In a multicenter study on the outcome of extremely premature infants at 22–28 weeks in the US from 1993 to 2012, ROP of stage 3 or higher increased from 13% of infants (124 of 941) in 1993 to 19% (262 of 1385) in 2003 but decreased to 11% of infants (160 of 1,509) by 2012 (20). These changes can be attributed to a better understanding of the risk factors and pathogenesis of ROP, leading to improvements in perinatal and neonatal care, which improve the primary prevention of severe ROP in EP infants (7). A study conducted by a single center in Hong Kong, China, on the trends in ROP in preterm infants <32 weeks from 2006 to 2015 found a decreasing trend in the incidence of type 1 ROP (severe ROP requiring treatment) for the subgroup with gestational age <28 weeks, although it did not reach statistical significance (21). However, our data indicates an increasing trend in the incidence of severe ROP. One reason for the increasing severe ROP might be that more EP infants survived, similar to the “second epidemic” of severe ROP in developed countries. The EP infants born between 26 and 27 weeks accounted for nearly 90% of the study population, and significant increase of survival rate has been observed among these infants. The overall survival rate of EP infants in our center significantly increased by 17.7% from P1 to P2, and the survival rate in P2 was slightly increased compared with that in the multicenter study in China from 2013–2014 (79 vs. 68.2%). The rising survival rate suggested an improved management of these extremely preterm infants both prenatally, such as an increasing use of antenatal steroids and the post-natal period. In addition, the overall increase in the number of EP infants, which has almost tripled from 34 in 2010 to 100 in 2019, leading more infants screened for ROP, which could also attribute to the increase of ROP incidence in the latter half of the study period.

Moreover, our study showed a higher incidence of ROP treatment for every specific GA stratum than in high-income countries. In Norway, 17 and 9% of preterm infants with a GA of 24 and 25 weeks were treated for ROP, respectively, and none of the infants with a GA > 25 weeks developed ROP requiring treatment in the period from 1999 to 2000 (22). In Switzerland, the incidences of ROP treatment were 14.5, 7.3, 2.7, and 1.1% among infants born at 24, 25, 26, and 27 weeks, respectively, in the 2006–2015 period (23). In our study population, the overall incidence of ROP treatment decreased from 25.0% at 24 weeks to 4.8% at 27 weeks as GA increased; however, it was much higher than the reported incidences in the Norwegian and Switzerland population-based study at each GA.

Efforts have been made in our unit during the past 10 years to improve overall outcomes of EP infants, as well as to reduce ROP.

First, a stricter strategy to use oxygen with blended air and oxygen and monitor oxygen saturation was adopted, although a high-target oxygen saturation (91–95%) has been adopted in this center since 2015. Percutaneous blood oxygen saturation and percutaneous carbon dioxide monitoring were used for EP infants with invasive ventilation to accurately adjust ventilator parameters and avoid excessive fluctuation of blood oxygen saturation, which has been shown in both animal and human clinical studies to increase the risk of severe ROP (24).

Second, the introduction of new non-invasive ventilation strategies in our center since 2015 reduced the proportion of EP infants requiring prolonged MV, which is among the most frequently identified risk factors for ROP. A study conducted in a single center in Spain that included 228 infants with a mean GA of 28.83 ± 2.03 weeks demonstrated that infants with a longer MV time had a higher risk of ROP treatment (an increase in risk of 8.1% for each additional day) (25).

Third, caffeine has been routinely used in EP infants since 2014, which may contributing to early extubation and reduction in severe ROP (26).

Last, the rate of exclusive expressed breastmilk feeding among EP infants increased significantly from 0.7% in P1 to 49.2% in P2 since the establishment of a hospital-based human milk bank in our NICU in 2017. Meta-analyses has shown that human milk is strongly associated with ROP protection (27–29).

Unfortunately, despite of these efforts, the incidence of severe ROP in our center is still on the rise. Except for possible influence from the increasing survival rate of EP infants, practice differences between our unit and developed countries must be examined further in detail and to facilitate targeted quality improvement initiatives.

There are some limitations of our study. First, the retrospective nature of this study inevitably generates inconsistencies in the data, although every effort was made to exclude subjects with incomplete clinical data. Second, our data showed that a significant proportion of infants were taken home by their parents against medical advice. Concerns about adverse outcomes and costs may be the main reason for the negative treatment attitude toward extremely preterm infants. Of these, the vast majority (88.4%, 76 of 86) abandoned care before ROP screening. The infants in this population may survive if treatment was not withdrawn; however, the risk of developing severe ROP needing treatment may be higher. Therefore, the actual incidence of ROP requiring treatment may be underestimated. However, there was no significant difference in the rate of withdrawal care during the 10-year period, which did not affect the trends analysis of ROP incidence. Third, this study was mainly a hospital-based study rather than a population-based study, but our unit is one of the largest NICUs in China. Our study provided information on the trends in ROP incidence in an extremely preterm Chinese population. The results serve as a baseline for future multicenter, prospective trials among Chinese populations.

In conclusion, we observed an increasing trend in the incidence of ROP in EP infants across the 10-year period in one of the largest NICUs in China. The increased survival rate and the use of high-target oxygen saturation in the later period may partly explain this trend, although substantial changes in the NICU protocols and practices have also evolved during this period. More evidence-based management is needed to reduce the incidence of ROP among EP infants.

## Data Availability Statement

The raw data supporting the conclusions of this article will be made available by the authors, without undue reservation.

## Ethics Statement

The studies involving human participants were reviewed and approved by the Ethics Committee of the Children's Hospital of Fudan University. Written informed consent to participate in this study was provided by the participants' legal guardian/next of kin.

## Author Contributions

YD, CC, and SZ: study design. YD, LZ, YZ, and SZ: the collection, analysis, and interpretation of data. YD, LZ, CC, and SZ: manuscript preparation. YD, LZ, YZ, CC, and SZ: final approval.

## Conflict of Interest

The authors declare that the research was conducted in the absence of any commercial or financial relationships that could be construed as a potential conflict of interest.

## Publisher's Note

All claims expressed in this article are solely those of the authors and do not necessarily represent those of their affiliated organizations, or those of the publisher, the editors and the reviewers. Any product that may be evaluated in this article, or claim that may be made by its manufacturer, is not guaranteed or endorsed by the publisher.

## References

[1] Zhu Z Yuan L Wang J Li Q Yang C Gao X. Mortality and morbidity of infants born extremely preterm at tertiary medical centers in China From 2010 to 2019. JAMA Netw Open. (2021) 4:e219382. 10.1001/jamanetworkopen.2021.9382 33974055 PMC8114138

[2] Kim SJ Port AD Swan R Campbell JP Chan RVP Chiang MF. Retinopathy of prematurity: a review of risk factors and their clinical significance. Surv Ophthalmol. (2018) 63:618–37. 10.1016/j.survophthal.2018.04.002 29679617 PMC6089661

[3] Hellgren KM Tornqvist K Jakobsson PG Lundgren P Carlsson B Källén K. Ophthalmologic outcome of extremely preterm infants at 6.5 years of age: extremely preterm infants in Sweden study (EXPRESS). JAMA Ophthalmol. (2016) 134:555–62. 10.1001/jamaophthalmol.2016.0391 27010699

[4] Molloy CS Anderson PJ Anderson VA Doyle LW. The long-term outcome of extremely preterm (<28 weeks' gestational age) infants with and without severe retinopathy of prematurity. J Neuropsychol. (2016) 10:276–94. 10.1111/jnp.12069 25809467

[5] Glass TJA Chau V Gardiner J Foong J Vinall J Zwicker JG. Severe retinopathy of prematurity predicts delayed white matter maturation and poorer neurodevelopment. Arch Dis Child Fetal Neonatal Ed. (2017) 102:F532–7. 10.1136/archdischild-2016-312533 28536205

[6] Hellstrom A Smith LE Dammann O. Retinopathy of prematurity. Lancet. (2013) 382:1445–57. 10.1016/S0140-6736(13)60178-6 23782686 PMC4389630

[7] Gilbert C Fielder A Gordillo L Quinn G Semiglia R Visintin P. Characteristics of infants with severe retinopathy of prematurity in countries with low, moderate, and high levels of development: implications for screening programs. Pediatrics. (2005) 115: e518–25. 10.1542/peds.2004-1180 15805336

[8] Darlow BA Lui K Kusuda S Reichman B Håkansson S Bassler D. International network for evaluating outcomes of neonates. International variations and trends in the treatment for retinopathy of prematurity. Br J Ophthalmol. (2017) 101:1399–404. 10.1136/bjophthalmol-2016-310041 28270489

[9] Gilbert C. Retinopathy of prematurity: a global perspective of the epidemics, population of babies at risk and implications for control. Early Hum Dev. (2008) 84:77–82. 10.1016/j.earlhumdev.2007.11.009 18234457

[10] Bowe T Nyamai L Ademola-Popoola D Amphornphruet A Anzures R Cernichiaro-Espinosa LA. The current state of retinopathy of prematurity in India, Kenya, Mexico, Nigeria, Philippines, Romania, Thailand, and Venezuela. Digit J Ophthalmol. (2019) 25:49–58. 10.5693/djo.01.2019.08.002 32076388 PMC7001648

[11] Sai Kiranmayee P Kalluri V. India to gear up to the challenge of “third epidemic” of retinopathy of prematurity in the world. Indian J Ophthalmol. (2019) 67:726–31. 10.4103/ijo.IJO_700_18 31124480 PMC6552629

[12] Ministry of Public Health of China. Guidelines on oxygenation policies and on prevention and treatment of retinopathy of prematurity. Chinese Nursing Management. (2004) 4:5–5, 64. 10.3969/j.issn.1672-1756.2004.04.001

[13] Colaboration Group of Retinopathy of Premature Infants. Multicenter survey on the clinical features and fundus lesions of retinopathy in premature infants in mainland China. Chin J Evid Based Pediat. (2015) 10:161–5. 10.3969/j.issn.1673-5501.2015.03.001

[14] Saugstad OD Aune D. Optimal oxygenation of extremely low birth weight infants: a meta-analysis and systematic review of the oxygen saturation target studies. Neonatology. (2014) 105:55–63. 10.1159/000356561 24247112

[15] International Committee for the Classification of Retinopathy of Prematurity. The International Classification of Retinopathy of Prematurity Revisited. Arch Ophthalmol. (2005) 123:991–9. 10.1001/archopht.123.7.991 16009843

[16] Chinese Medical Association Ophthalmology Branch Fundus Study Group. Guidelines for screening retinopathy of premature infants in China (2014). Chin J Ophthalmol. (2014) 50:933–5. 10.3760/cma.j.issn.0412-4081.2014.12.017

[17] Early Treatment For Retinopathy Of Prematurity Cooperative Group. Revised indications for the treatment of retinopathy of prematurity: results of the early treatment for retinopathy of prematurity randomized trial. Arch Ophthalmol. (2003) 121:1684–94. 10.1001/archopht.121.12.1684 14662586

[18] Ballard JL Khoury JC Wedig K Wang L Eilers-Walsman BL Lipp R. New Ballard Score, expanded to include extremely premature infants. J Pediatr. (1991) 119:417–23. 10.1016/S0022-3476(05)82056-6 1880657

[19] Zhu L Zhang R Zhang S Shi W Yan W Wang X. Chinese neonatal birth weight curve for different gestational age. Chin J Pediatr. (2015) 53:97–103. 10.3760/cma.j.issn.0578-1310.2015.02.007 25876683

[20] Stoll BJ Hansen NI Bell EF Walsh MC Carlo WA Shankaran S. Trends in care practices, morbidity, and mortality of extremely preterm neonates, 1993–2012. JAMA. (2015) 314:1039–51. 10.1001/jama.2015.10244 26348753 PMC4787615

[21] Chow PPC Yip WWK Ho M Lok JYC Lau HHW Young AL. Trends in the incidence of retinopathy of prematurity over a 10-year period. Int Ophthalmol. (2019) 39:903–9. 10.1007/s10792-018-0896-0 29907928

[22] Markestad T Kaaresen PI Rønnestad A Reigstad H Lossius K Medbø S. Early death, morbidity, and need of treatment among extremely premature infants. Pediatrics. (2005) 115:1289–98. 10.1542/peds.2004-1482 15867037

[23] Gerull R Brauer V Bassler D Laubscher B Pfister RE Nelle M. Swiss Incidence of Retinopathy of Prematurity (ROP) and ROP treatment in Switzerland 2006–2015: a population-based analysis. Arch Dis Child Fetal Neonatal Ed. (2018) 103:F337–42. 10.1136/archdischild-2017-313574 28916563

[24] Hartnett ME Penn JS. Mechanisms and management of retinopathy of prematurity. N Engl J Med. (2013) 368:1162–3. 10.1056/NEJMra1208129 23514302

[25] Chaves-Samaniego MJ García Castejón M Chaves-Samaniego MC Solans Perez Larraya A Ortega Molina JM Muñoz Hoyos A. Risk calculator for retinopathy of prematurity requiring treatment. Front Pediatr. (2020) 8:529639. 10.3389/fped.2020.529639 33042928 PMC7530187

[26] Martin A Ghadge A Manzoni P Lui K Brown R Tarnow-Mordi W. Lactoferrin Infant Feeding Trial (LIFT): a randomized trial of adding lactoferrin to the feeds of very-low birthweight babies prior to hospital discharge. BMJ Open (2018) 8:e023044. 10.1136/bmjopen-2018-023044 30282685 PMC6169746

[27] Zhou J Shukla VV John D Chen C. Human milk feeding as a protective factor for retinopathy of prematurity: a meta-analysis. Pediatrics. (2015) 136:e1576–86. 10.1542/peds.2015-2372 26574589

[28] Fang JL Sorita A Carey WA Colby CE Murad MH Alahdab F. Interventions to prevent retinopathy of prematurity: a meta-analysis. Pediatrics. (2016) 137:e20153387. 10.1542/peds.2015-3387 26962240

[29] Bharwani SK Green BF Pezzullo JC Bharwani SS Bharwani SS Dhanireddy R. Systematic review and meta-analysis of human milk intake and retinopathy of prematurity: a significant update. J Perinatol. (2016) 36:913–20. 10.1038/jp.2016.98 27416321

